# Supplementary figures and images for: Identification of IMC43, a novel IMC protein that collaborates with IMC32 to form an essential daughter bud assembly complex in Toxoplasma gondii
Source: PLoS Pathog. 2023 Oct 2;19(10):e1011707. doi: 10.1371/journal.ppat.1011707 (PMC10569561; doi:10.1371/journal.ppat.1011707)

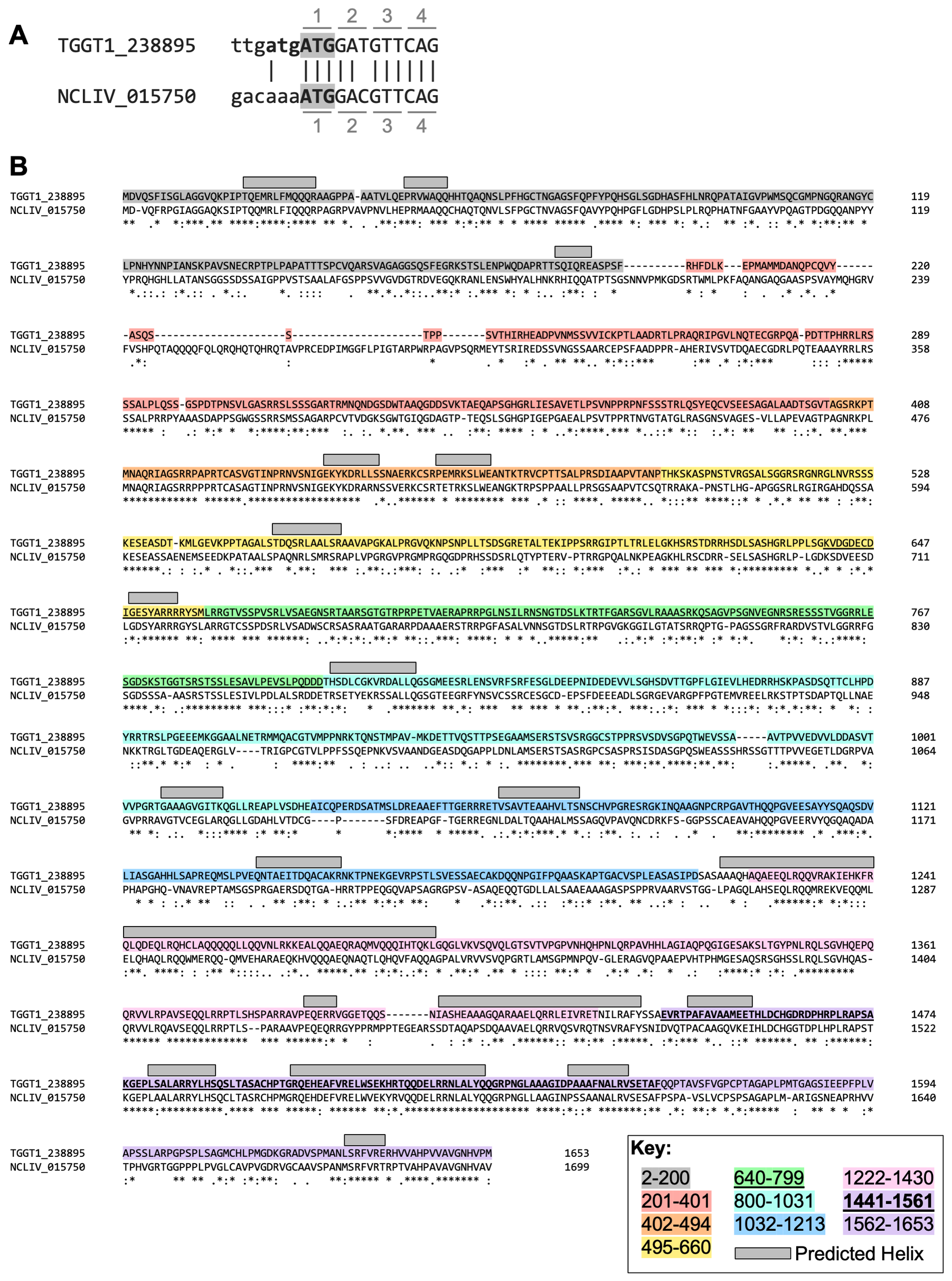

Supplement: S1 Fig — A) ToxoDB reports TGGT1_238895 as a 1,654 amino acid protein, with the first two residues being methionine. Alignment of TGGT1_238895 with its N. caninum homolog NCLIV_015750 show that the first methionine (lowercase, bold) in TGGT1_238895 is not conserved. The second methionine (uppercase, bold) and much of the N-terminus is highly conserved. The second methionine is also a more favorable start codon based on the T. gondii consensus translation initiation sequence (A at position -3, G at position +4) [77]. We therefore determined that the protein is likely to start at the second methionine, resulting in TGGT1_238895 encoding a 1,653 amino acid protein. Residues are numbered accordingly in this study. B) The amino acid sequence of IMC43 (TGGT1_238895) was aligned to its N. caninum ortholog NCLIV_015750 using ClustalO 1.2.4. Alpha-helices predicted by PSIPRED are shown above their corresponding sequences. Regions chosen for the deletion series are highlighted. (TIF) [file ppat.1011707.s001.tif]

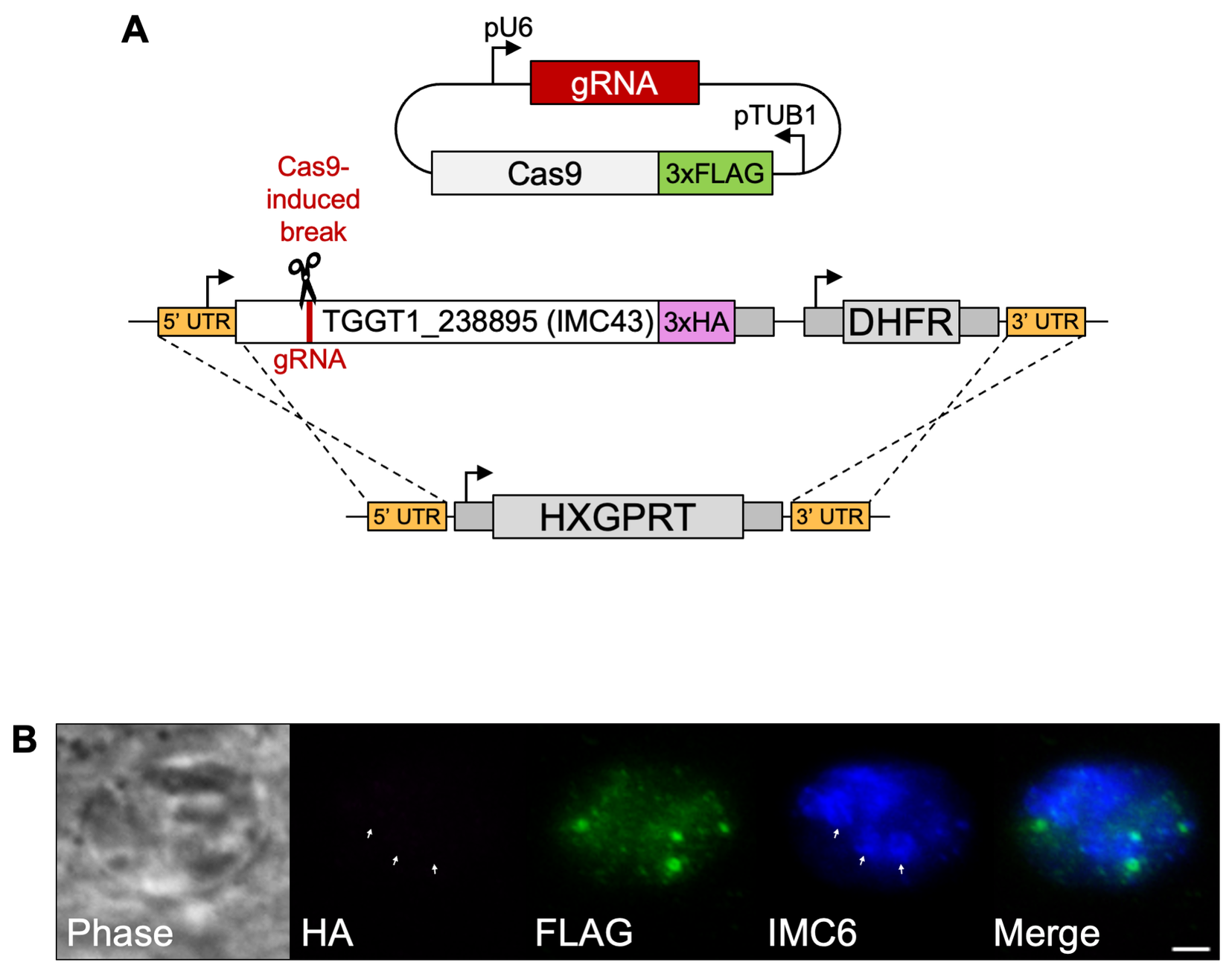

Supplement: S2 Fig — A) Diagram showing the strategy used to genetically disrupt IMC43 in an IMC433xHA parental line. B) IFA showing that FLAG-positive/HA-negative parasites with severe morphological defects were observed 30 hours after transfection, indicating successful ablation of the target gene. The Δimc43 parasites were rapidly lost from the population and could not be recovered. Arrows point to daughter buds lacking HA staining. Magenta = anti-HA, Green = anti-FLAG detecting Cas93xFLAG, Blue = anti-IMC6. Scale bar = 2 μm. (TIF) [file ppat.1011707.s002.tif]

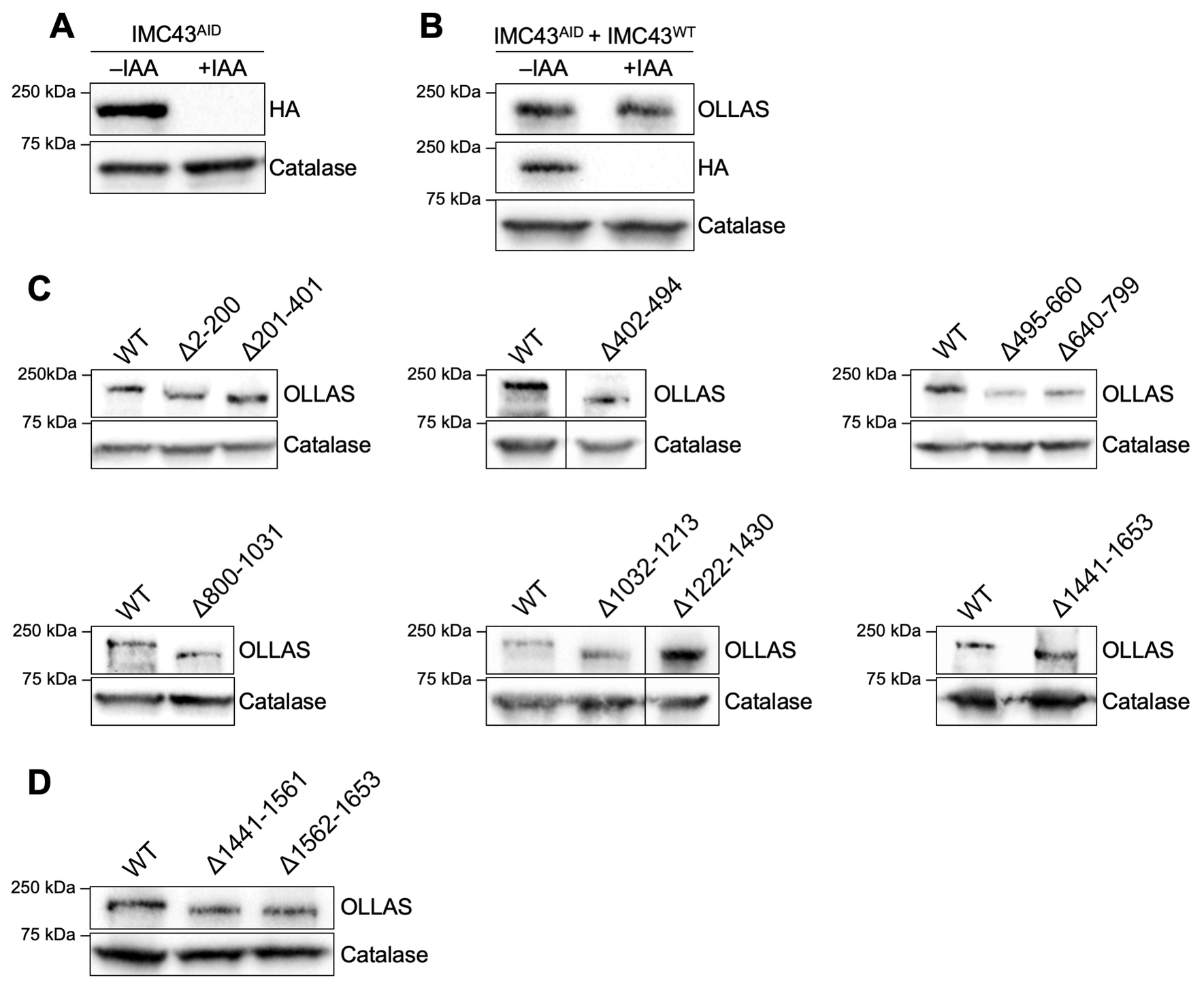

Supplement: S3 Fig — A) Western blot showing efficient depletion of IMC43AID after four hours of IAA treatment. Parasites were grown intracellularly for 24 hours prior to adding IAA. Catalase is used as a loading control. B) Western blot of IMC43AID + IMC43WT parasites after four hours of IAA treatment. IMC43WT expresses at equal levels -/+ IAA. Catalase is used as a loading control. C) Western blots comparing expression levels of IMC43WT with the IMC43 deletion constructs shown in Fig 4A. Catalase was used as a loading control. D) Western blots comparing expression levels of IMC43WT with IMC43Δ1441–1561 and IMC43Δ1562–1653. Catalase was used as a loading control. (TIF) [file ppat.1011707.s003.tif]

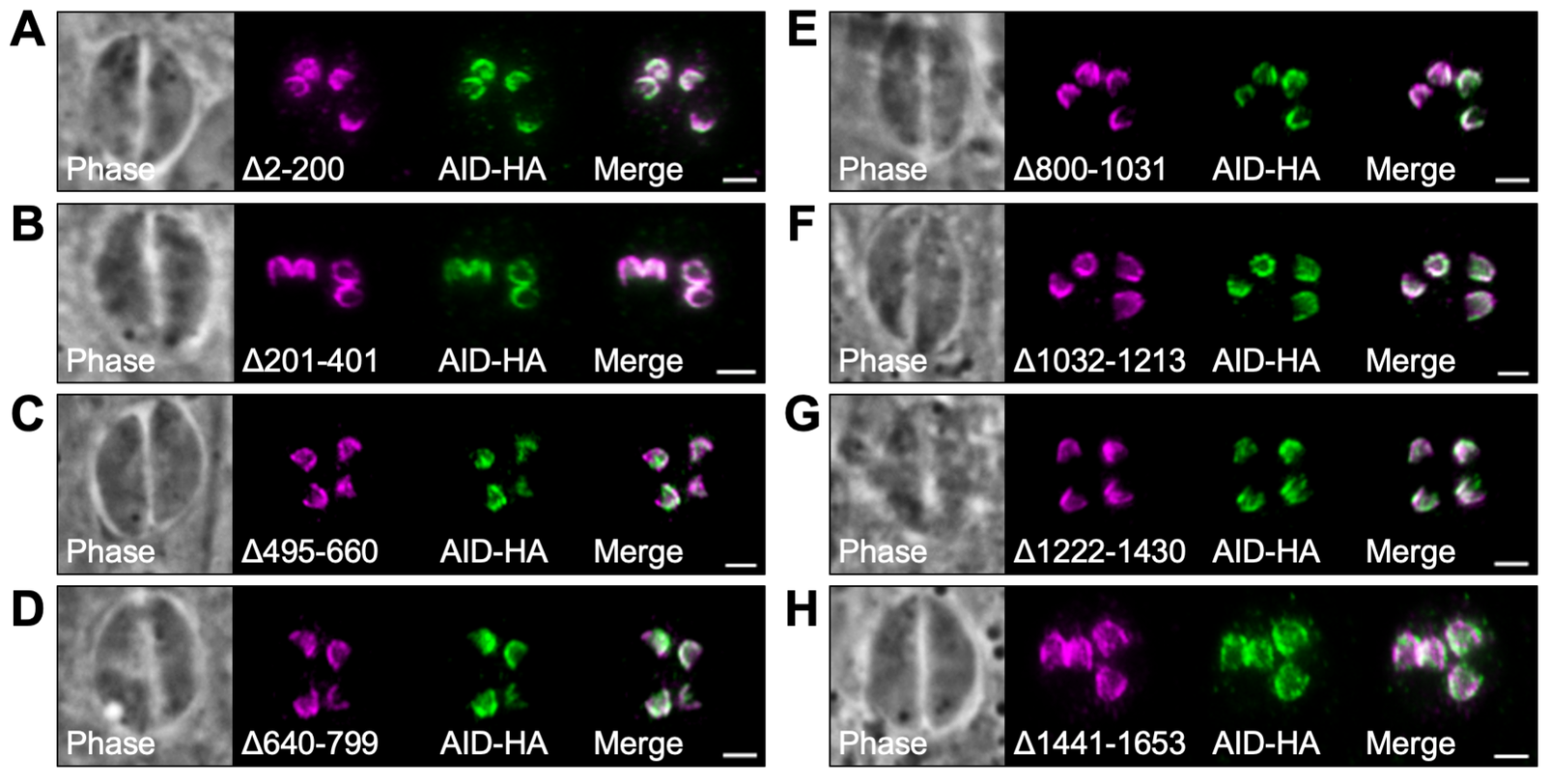

Supplement: S4 Fig — IFAs showing that eight of the IMC43 deletion constructs colocalize with IMC43AID. Magenta = anti-OLLAS detecting IMC43 deletion constructs, Green = anti-HA detecting IMC43AID. Scale bars = 2 μm. (TIF) [file ppat.1011707.s004.tif]

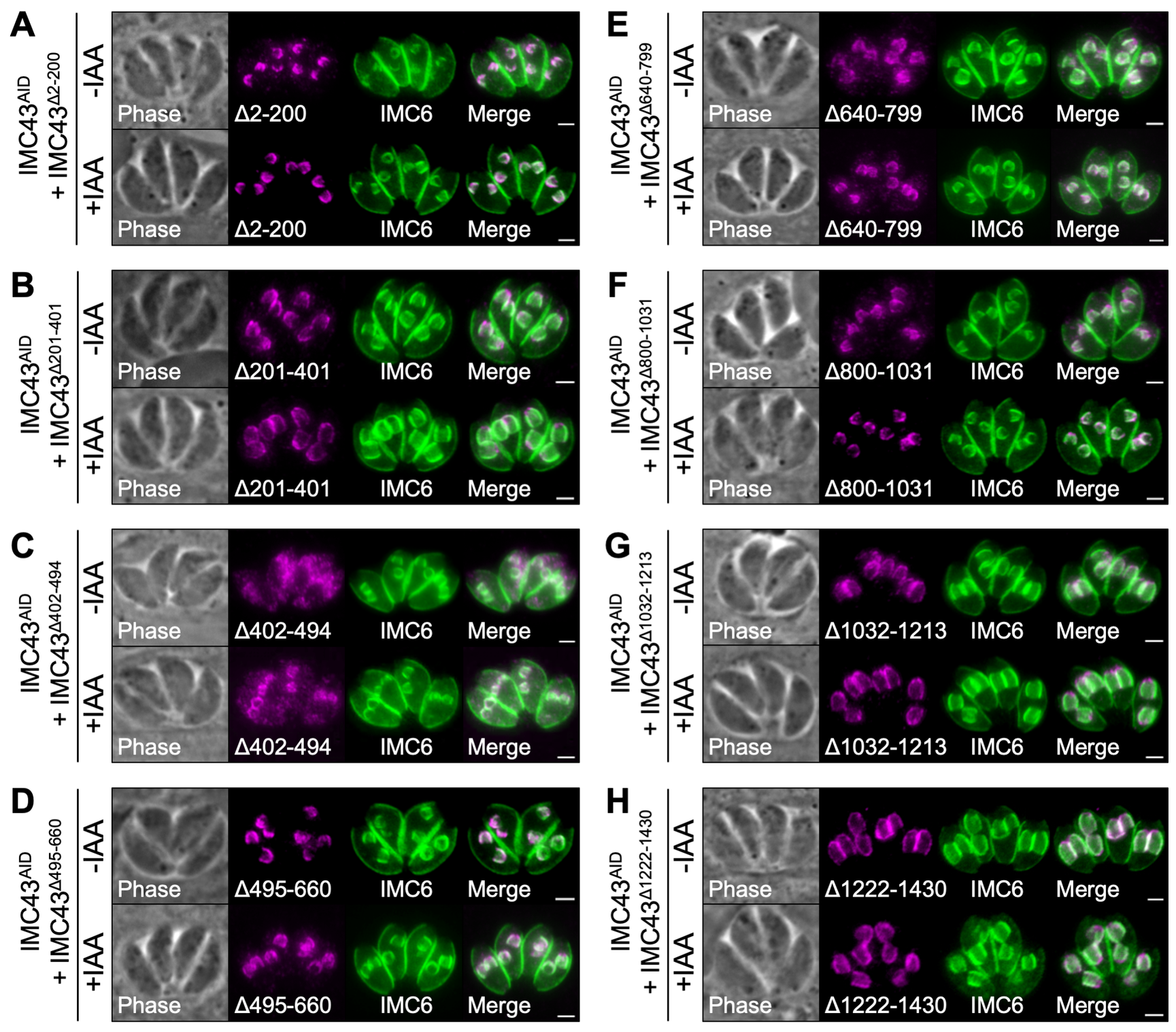

Supplement: S5 Fig — IFAs showing that eight of the IMC43 deletion constructs fully rescue the morphological and replication defects caused by depletion of IMC43. Magenta = anti-OLLAS detecting IMC43 deletion constructs, Green = anti-IMC6. Scale bars = 2 μm. (TIF) [file ppat.1011707.s005.tif]

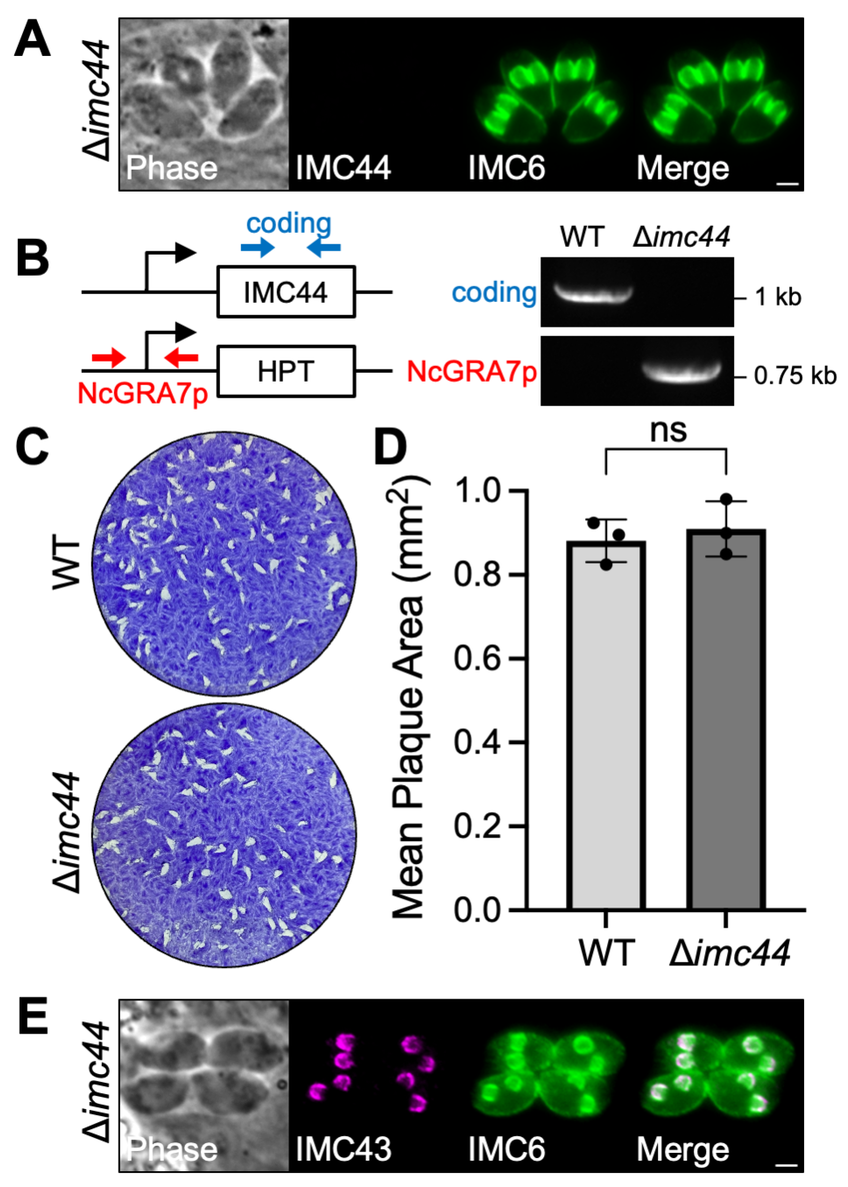

Supplement: S6 Fig — A) The endogenous locus for IMC44 was disrupted in the IMC443xMyc parent strain. IFA of Δimc44 parasites confirms loss of IMC443xMyc signal. Magenta = anti-Myc, Green = anti-IMC6. B) PCR verification for genomic DNA of WT and Δimc44 parasites. Diagram indicates the binding location of primers used to amplify the IMC44 coding sequencing (blue arrows) and the site of recombination for the knockout (red arrows). C) Plaque assays of WT and Δimc44 parasites. D) Quantification of plaque size for plaque assays shown in panel D. Statistical significance was determined using a two-tailed t test (ns = not significant). E) IFA showing normal localization of IMC43 in Δimc44 parasites. Magenta = anti-Ty detecting IMC432xStrep3xTy, Green = anti-IMC6. Scale bars = 2 μm. (TIF) [file ppat.1011707.s006.tif]

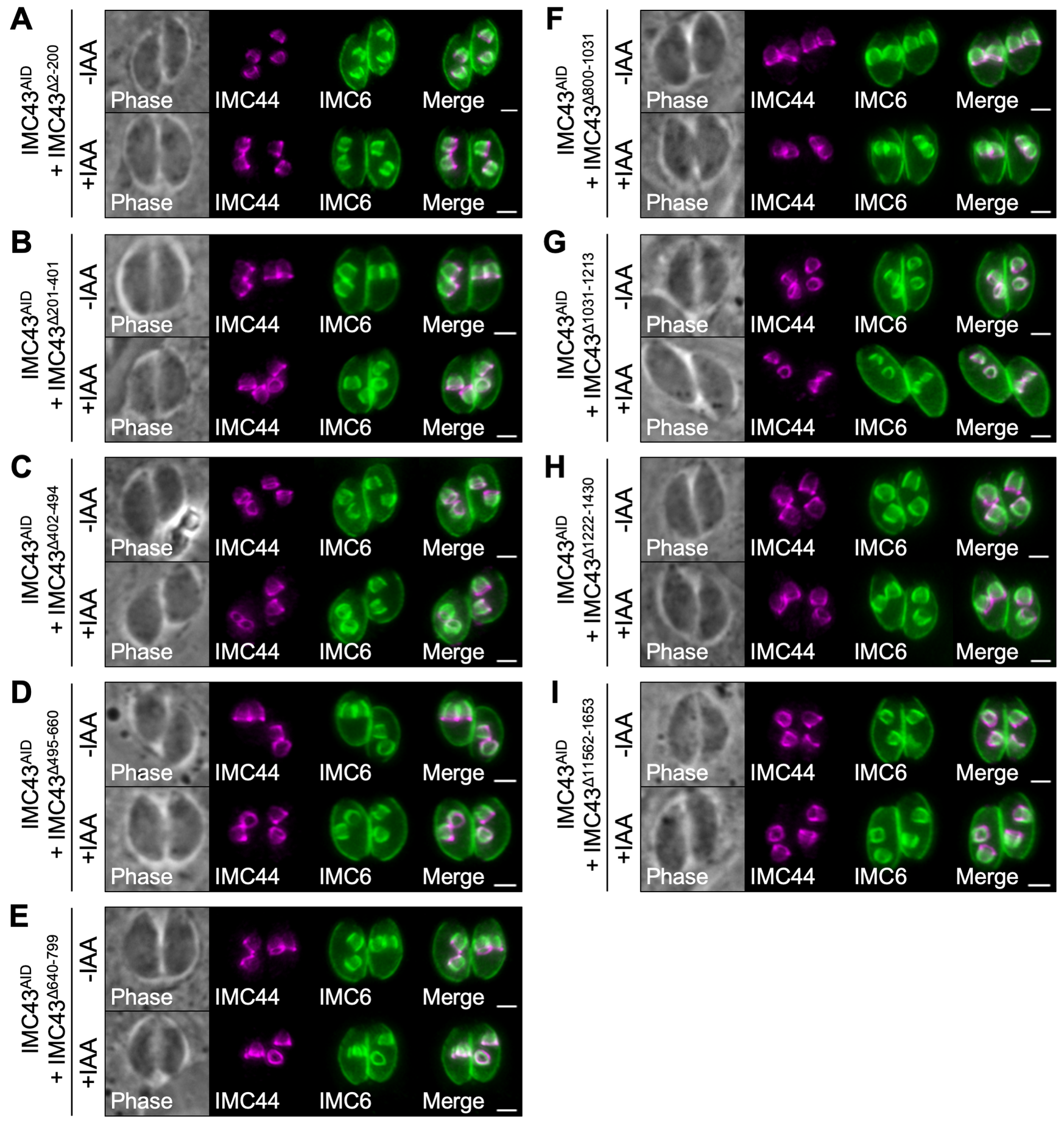

Supplement: S7 Fig — IFAs showing the localization of IMC44 in nine of the IMC43 deletion lines. All nine shown in this figure rescue the mislocalization of IMC44. Magenta = anti-Myc detecting IMC443xMyc, Green = anti-IMC6. Scale bars = 2 μm. (TIF) [file ppat.1011707.s007.tif]

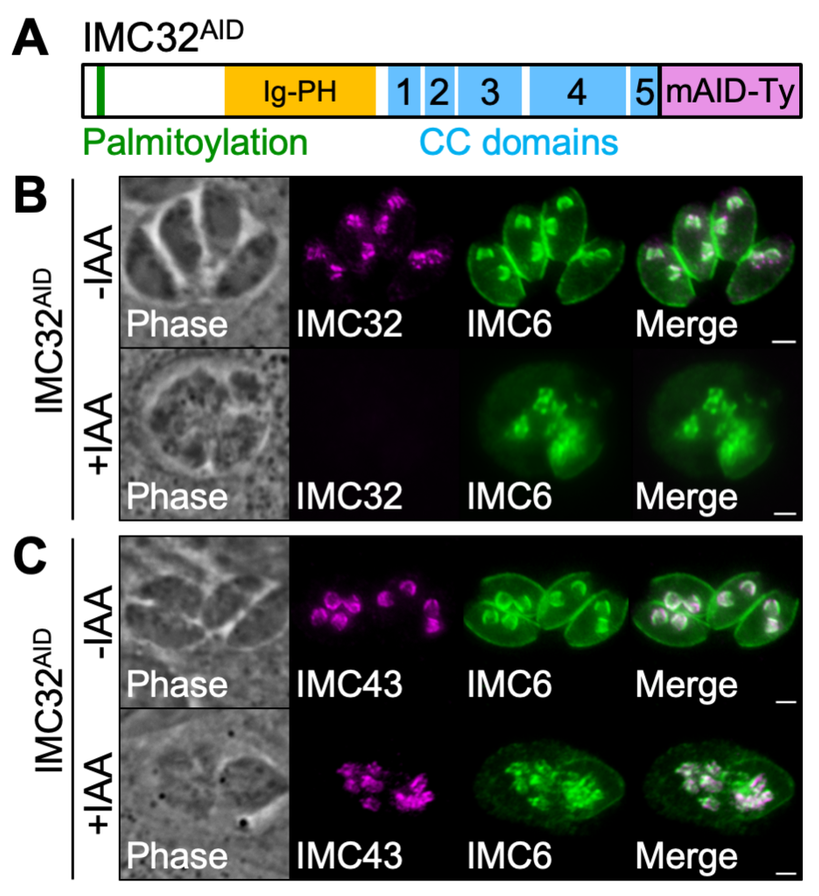

Supplement: S8 Fig — A) An mAID-3xTy degron tag was fused to the C-terminus of IMC32 in a TIR1-expressing strain to facilitate proteasomal degradation upon treatment with IAA. B) IFA of IMC32AID parasites after 24 hours of growth -/+ IAA. Depletion of IMC32 results in morphological and replication defects as previously described [27]. Magenta = anti-Ty detecting IMC32AID, Green = anti-IMC6. C) IFA showing the localization of IMC43 in IMC32AID parasites after 24 hours of growth -/+ IAA. IMC43 is unaffected by depletion of IMC32. Magenta = anti-HA detecting IMC433xHA, Green = anti-IMC6. Scale bars = 2 μm. (TIF) [file ppat.1011707.s008.tif]

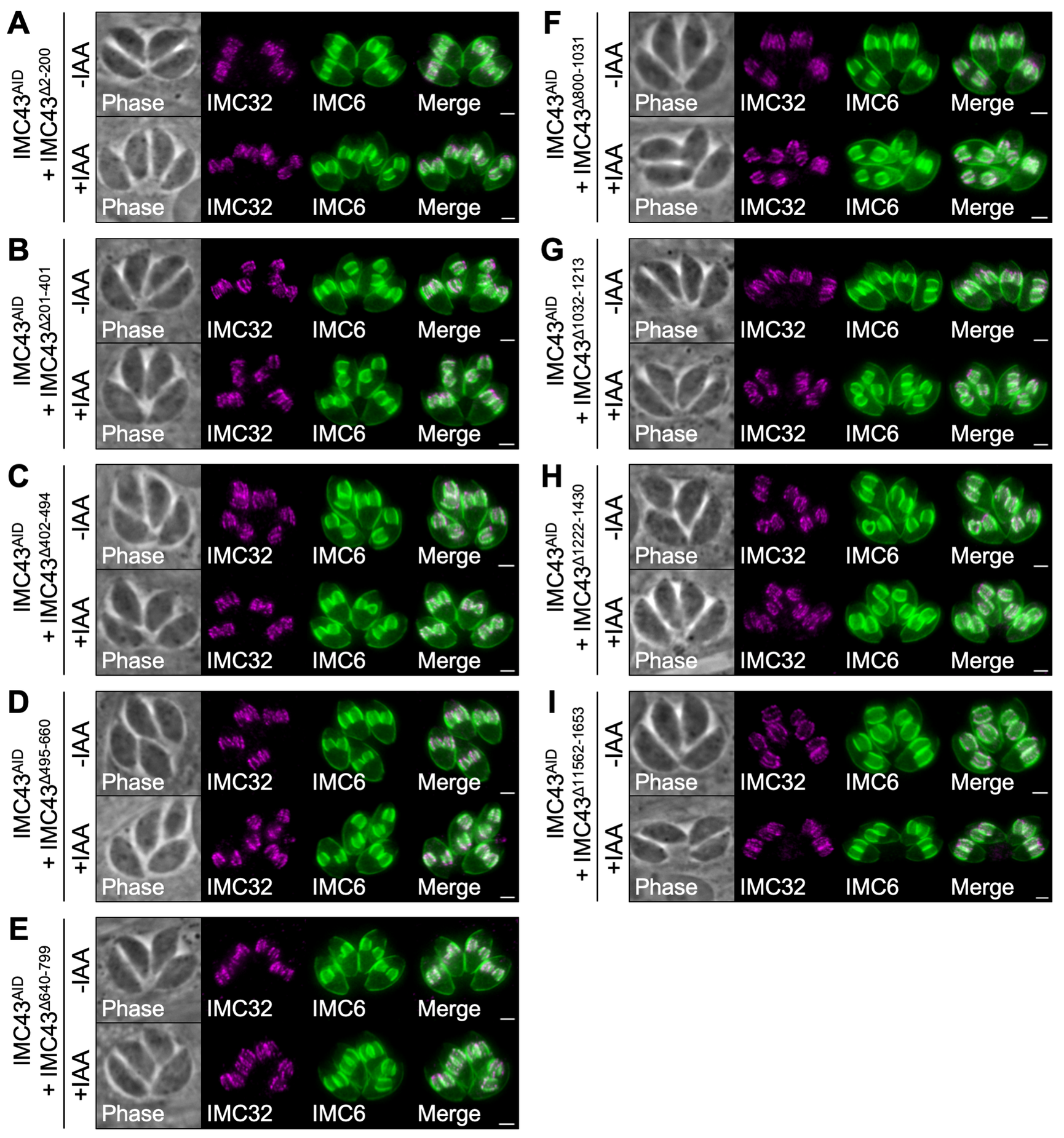

Supplement: S9 Fig — IFAs showing the localization of IMC32 in nine of the IMC43 deletion lines. All nine shown in this figure rescue the mislocalization of IMC32. Magenta = anti-V5 detecting IMC323xV5, Green = anti-IMC6. Scale bars = 2 μm. (TIF) [file ppat.1011707.s009.tif]
